# Detecting Coronary Artery Disease from Computed Tomography Images Using a Deep Learning Technique

**DOI:** 10.3390/diagnostics12092073

**Published:** 2022-08-26

**Authors:** Abdulaziz Fahad AlOthman, Abdul Rahaman Wahab Sait, Thamer Abdullah Alhussain

**Affiliations:** 1 Department of Documents and Archive, Center of Documents and Administrative Communication, King Faisal University, P.O. Box 400, Al Hofuf 31982, Al-Ahsa, Saudi Arabia; 2 Programming and Electronic Services Department, Admission and Registration Deanship, King Faisal University, P.O. Box 400, Al Hofuf 31982, Al-Ahsa, Saudi Arabia

**Keywords:** coronary artery disease, deep learning, machine learning, cardiopulmonary disease, faster CNN

## Abstract

In recent times, coronary artery disease (CAD) has become one of the leading causes of morbidity and mortality across the globe. Diagnosing the presence and severity of CAD in individuals is essential for choosing the best course of treatment. Presently, computed tomography (CT) provides high spatial resolution images of the heart and coronary arteries in a short period. On the other hand, there are many challenges in analyzing cardiac CT scans for signs of CAD. Research studies apply machine learning (ML) for high accuracy and consistent performance to overcome the limitations. It allows excellent visualization of the coronary arteries with high spatial resolution. Convolutional neural networks (CNN) are widely applied in medical image processing to identify diseases. However, there is a demand for efficient feature extraction to enhance the performance of ML techniques. The feature extraction process is one of the factors in improving ML techniques’ efficiency. Thus, the study intends to develop a method to detect CAD from CT angiography images. It proposes a feature extraction method and a CNN model for detecting the CAD in minimum time with optimal accuracy. Two datasets are utilized to evaluate the performance of the proposed model. The present work is unique in applying a feature extraction model with CNN for CAD detection. The experimental analysis shows that the proposed method achieves 99.2% and 98.73% prediction accuracy, with F1 scores of 98.95 and 98.82 for benchmark datasets. In addition, the outcome suggests that the proposed CNN model achieves the area under the receiver operating characteristic and precision-recall curve of 0.92 and 0.96, 0.91 and 0.90 for datasets 1 and 2, respectively. The findings highlight that the performance of the proposed feature extraction and CNN model is superior to the existing models.

## 1. Introduction

Coronary artery disease (CAD) has recently become regarded as one of the most dangerous and life-threatening chronic diseases [1]. Blockage and narrowing of the coronary arteries is the primary cause of heart failure. The coronary arteries must be open to provide the heart with adequate blood [2,3,4]. According to a recent survey, the United States has the highest heart disease prevalence and the highest ratio of heart disease patients [5]. Shortness of breath, swelling feet, fatigue, and other symptoms of heart disease are among the most frequent. CAD is the most common type of heart disease, which can cause chest discomfort, stroke, and heart attack. Besides heart disease, there are heart rhythm issues, congestive heart failure, congenital heart disease, and cardiovascular disease [6].

Traditional methods of investigating cardiac disease are complex [7,8,9,10]. The lack of medical diagnostic instruments and automated systems makes pulmonary heart disease detection and treatment challenging in developing nations. However, to reduce the impact of CAD, an accurate and appropriate diagnosis of cardiac disease is necessary. Developing countries experience an alarming rise in the number of people dying from heart disease [11,12,13,14,15,16]. According to WHO, CAD is the most frequent type of heart disease, claiming the lives of 360,900 individuals globally in 2019 [17]. The sum accounts for nearly 30% of all deaths worldwide. The number of persons who are victimized is increasing exponentially. Multiple risk factors are involved in the CAD prediction. Thus, healthcare centers require a tool to detect CAD at earlier stages. The recent developments in CNN models enable researchers to develop a prediction model for CAD. However, CNN’s structure is complex and needs an excellent graphical processing unit (GPU) to process complex images.

Among conventional approaches, analytical angiography is considered one of the most accurate procedures for detecting heart abnormalities. The disadvantages of angiography include the expensive cost, various side effects, and the need for a high level of technological competence [18]. Due to human error, conventional methods often yield inaccurate diagnoses and take longer to complete. In addition, it is a costly and time-consuming method for diagnosing disease and requires considerable processing.

Artificial intelligence (AI) applications have been increasingly included in clinical diagnostic systems during the last three decades to improve their accuracy. Data-driven decision-making using AI algorithms has been increasingly common in the CAD field in recent years [19]. The diagnostic accuracy can be improved by automating and standardizing the interpretation and inference processes. AI-based systems can help speed up decision-making. Healthcare centers can obtain, evaluate, and interpret data from these emerging technologies and facilitate better patient service [20]. The raw data can significantly affect the quality and performance of AI approaches. As a result, extensive collaboration between AI engineers and clinical professionals is required to improve the quality of diagnosis [21]. The recent CAD detection technique is based on images. Faster predictions can be made for clinicians and computer scientists by deleting irrelevant features. The key features representing the crucial part of CAD decide the performance of the AI techniques [22]. Many studies use deep learning (DL) to determine the existence of CAD.

Convolutional neural networks (CNN) are becoming increasingly popular in medical image processing. CNN was initially demonstrated in medical image analysis in the work of [23] for lung nodule diagnosis. Numerous medical imaging techniques are based on this concept [24,25,26,27]. Using a pre-trained network as a feature generator and fine-tuning a pre-trained network to categorize medical pictures are two strategies to transmit the information stored in the pre-trained CNNs. Standard networks can be divided into multiple classes as pre-trained medical image analysis models. Kernels with large receptive fields are used in the higher layers near the input, while smaller kernels are used in the deeper levels. Among the networks in this group, AlexNet is the most widely used and has many applications in medical image processing [28,29,30,31].

Deep learning networks are advanced AI techniques and have gained popularity in the medical field. The first network in this category was GoogleNet [32,33,34,35,36]. However, there is a shortcoming in the existing methods, such as more computation time and high-end systems. In addition, the performance of the current CNN architectures is limited in terms of accuracy and F-Measure. In addition, literature is scarce related to integrating feature minimization and CAD techniques. Therefore, this study intends to develop a CNN-based classifier to predict CAD with high accuracy. The objective of the study is as follows:

- To build a CNN model to predict CAD from CT images.
- To improve the performance of CNN by reducing the number of features.

The research questions of the proposed study are:

Research Question-1 (RQ1): How to improve the performance of a CAD detection technique?

Research Question-2 (RQ2): How to evaluate the performance of a CAD detection technique?

The structure of the study is organized as follows: Section 2 presents the recent literature related to CNN and CAD. Section 3 outlines the methodology of the proposed research. Results and discussion are highlighted in Section 4. Finally, Section 5 concludes the study with its future improvement.

## 2. Literature Review

High-accuracy data-mining techniques can identify risk factors for heart disease. Studies on the diagnosis of CAD can be found in existing studies [1,2,3,4,5]. Artificial immune recognition system (AIRS), K nearest neighbor (KNN), and clinical data were used to develop a system for diagnosing CAD and achieved an accuracy rate of 87%.

The authors [1] developed and evaluated a deep-learning algorithm for diagnosing CAD based on facial photographs. Patients who underwent coronary angiography or CT angiography at nine Chinese locations participated in a multicenter cross-sectional study to train and evaluate a deep CNN to detect CAD using patient facial images. More than 5796 patients were included in the study and were randomly assigned to training and validation groups for algorithm development. According to the findings, a deep-learning algorithm based on facial photographs can help predict CAD.

According to a study [2], the combination of semi-upright and supine stress myocardial perfusion imaging with deep learning can be used to predict the presence of obstructive disease. The total perfusion deficit was calculated using standard gender and camera type limits. A study [3] employed interferometric OCT in cardiology to describe coronary artery tissues, yielding a resolution of between 10 and 20 μm. Using OCT, the authors [3] investigated the various deep learning models for robust tissue characterization to learn the various intracoronary pathological formations induced by Kawasaki disease. A total of 33 historical cases of intracoronary cross-sectional pictures from different pediatric patients with KD are used in the experimentation. The authors analyzed in-depth features generated from three pre-trained convolutional networks, which were then compared. Moreover, voting was conducted to determine the final classification.

The authors [6] used deep-learning analysis of the myocardium of the left ventricle to identify individuals with functionally significant coronary stenosis in rest coronary CT angiography (CCTA). There were 166 participants in the study who had invasive FFR tests and CCTA scans taken sequentially throughout time. Analyses were carried out in stages to identify patients with functionally significant stenosis of the coronary arteries.

Using deep learning, the researchers [7] investigated the accuracy of the automatic prediction of obstructive disease from myocardial perfusion imaging compared to the overall perfusion deficit. Single-photon emission computed tomography may be used to build deep convolutional neural networks that can better predict coronary artery disease in individual patients and individual vessels. Obstructive disease was found in 1018 patients (62%) and 1797 of 4914 (37%) arteries in this study. A larger area under the receiver-operating characteristic curve for illness prediction using deep learning than for total perfusion deficits. Myocardial perfusion imaging can be improved using deep learning compared to existing clinical techniques.

In the study [8], several deep-learning algorithms were used to classify electrocardiogram (ECG) data into CAD, myocardial infarction, and congestive heart failure. In terms of classification, CNNs and LSTMs tend to be the most effective architectures to use. This study built and verified a 16-layer LSTM model using a 10-fold cross-validation procedure. The accuracy of the classification was 98.5%. They claimed their algorithm might be used in hospitals to identify and classify aberrant ECG patterns.

Author [9] proposed an enhanced DenseNet algorithm based on transfer learning techniques for fundus medical imaging. Medical imaging data from the fundus has been the subject of two separate experiments. A DenseNet model can be trained from scratch or fine-tuned using transfer learning. Pre-trained models from a realistic image dataset to fundus medical images are used to improve the model’s performance. Fundus medical image categorization accuracy can be improved with this method, which is critical for determining a patient’s medical condition.

The study [10] developed and implemented a heterogeneous low-light image-enhancing approach based on DenseNet generative adversarial network. Initially, a generative adversarial network is implemented using the DenseNet framework. The generative adversarial network is employed to learn the feature map from low-light to normal-light images.

To overcome the gradient vanishing problem in deep networks, the DenseNet convolutional neural network with dense connections combines ResNet and Highway’s strengths [11,12]. As a result, all network layers can be directly connected through the DenseNet. Each layer of the network is directly related to the next layer. It is important to remember that each subsequent layer’s input is derived from the output of all preceding layers. The weak information transmitted in the deep network is the primary cause of the loss of gradients [13]. A more efficient way to reduce gradient disappearance and improve network convergence is to use the dense block design, in which each layer is directly coupled to input and loss [14].

The authors [15] employed a bright-pass filter and logarithmic transformation to improve the quality of an image. Simultaneous reflectance and illumination estimation (SRIE) was given a weighted variational model by the authors [16] to deal with the issue of overly enhanced dark areas. Authors [17] developed low light image enhancement by illumination map estimation (LIME), which simply estimates the illumination component. The reflection component of the image was calculated using local consistency and structural perception restrictions, and the output result was based on this calculation.

The study [18] used the Doppler signal and a neural network to gain the best possible CAD diagnosis. By combining the exercise test data with a support vector machine (SVM), the authors [19] achieved an accuracy of 81.46% in the diagnosis of coronary artery disease (CAD). By employing multiple neural networks, authors [20] achieved an accuracy of 89.01% for CAD diagnosis using the Cleveland dataset [21]. It is possible to forecast artery stenosis disease using various feature selection approaches, including CBA, filter, genetic algorithm, wrapper, and numerical and nominal attribute selection. Also, Ref. [22] uses a new feature creation method to diagnose CAD.

Inception-v3 [24] is an enhanced version of GoogleNet and is applied in medical image analysis. It categorizes knee images by training support vector machines using deep feature extraction from CaffeNets. Adults’ retinal fundus pictures were analyzed using a fine-tuned network to detect diabetic retinopathy [24]. Classification results utilizing fine-tuned networks compete with human expert performance [25]. Recent research has focused on applying deep learning techniques to segment retinal optical coherence tomography (OCT) images [26,27,28]. Combining CNN and graph search methods, OCT retinal images are segmented. Layer border classification probabilities are used in the Cifar-CNN architecture to partition the graph search layer [29,30].

Authors [31] proposed a deep learning technique to quantify and segment intraregional cystoid fluid using fuzzy CNN. Geographic atrophy (GA) segmentation using a deep network is the subject of another study [33]. An automated CAD detector was developed using a CNN with encoder–decoder architecture [34]. In another study, researchers employed GoogleNet to identify retinal diseases in OCT pictures [35].

Several grayscale features collected from echocardiogram pictures of regular and CAD participants were proposed in [36] as a computer-aided diagnosis approach. In [24], ECG data from routine and CAD participants was evaluated for HR signals. Various methods were used to examine the heart rate data, including non-linear analysis, frequency, and time-domain. They found that CAD participants’ heart rate signals were less erratic than normal subjects. The recent CNN models are widely applied in CAD diagnostics [36]. In [37], the authors proposed a model for identifying cardiovascular diseases and obtained a prediction accuracy of 96.75%. Ali Md Mamun et al. [38] argued that a simple supervised ML algorithm can predict heart disease with high accuracy. The authors [39] developed a biomedical electrocardiogram (ECG)-based ML technique for detecting heart disease. Jiely Yan et al. [40], proposed a model to predict ion channel peptide from the images. Table 1 outlines the features and limitations of the existing CNN models.

## 3. Research Methodology

**Figure 1:**
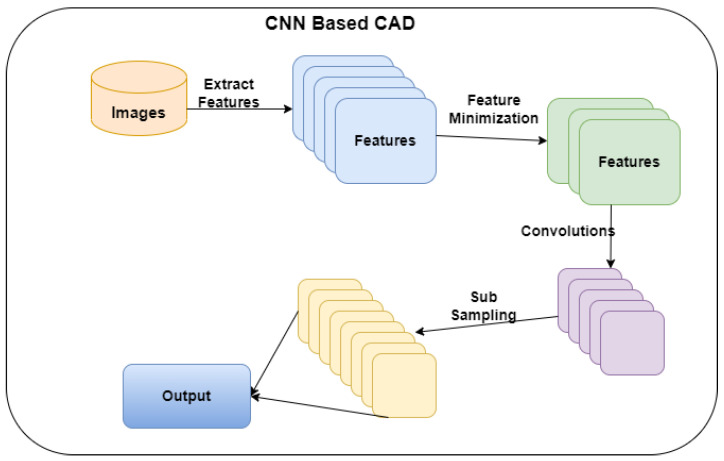
Proposed CNN network for CAD.

According to the research questions, the researchers developed a CNN architecture to predict positive CAD patients from CT images. Figure 1 presents the proposed architecture. Initially, the images are processed to extract the features. The CNN model treats the extracted features, generating output through an activation function. The following part of this section provides the information related to datasets, feature extraction, CNN construction, and evaluation metrics.

**Figure 2:**
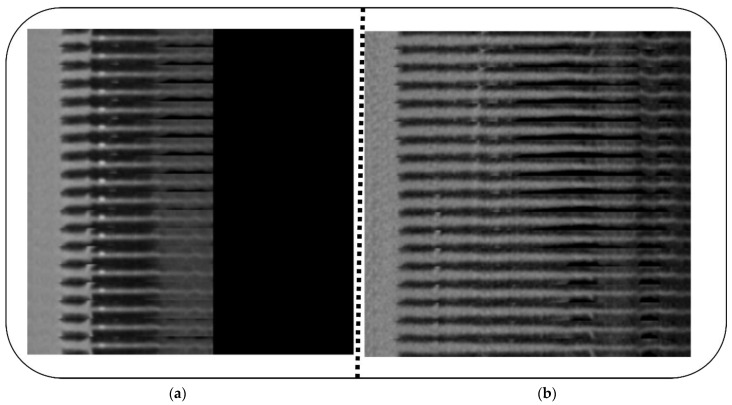
(**a**): Positive individual, (**b**): negative individual.

In this study, researchers employed two datasets of CT angiography images. The details of the datasets are as follows:

Dataset 1 [4] contains coronary artery image sets of 500 patients. A number of 18 views of the same straightened coronary artery are shown in each mosaic projection view (MPV). The Training–Validation–Test picture sets have a 3/1/1 ratio (300/100/100) with 50% normal and 50% sick cases for each patient in the subset. To improve modeling and dataset balance, 2364 (i.e., 394 × 6) artery pictures were obtained from the 300 training instances. Only 2304 images of the training dataset were augmented: 1. the standard component; 2. all the validation images; and 3. all the testing images. The balance was maintained in the validation dataset by randomly selecting one artery per normal case (50 images) and sick patient (50 images). Figure 2a,b outlines the CT images of positive and negative CAD patients.

**Figure 3:**
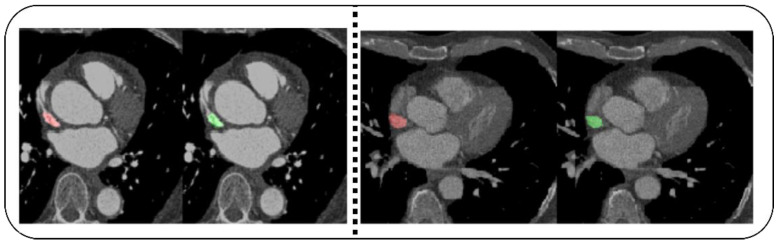
Superior vena cava images of individuals.

Dataset 2 [5] consists of CT angiography images of 200 patients. This dataset used images from a multicenter registry of patients who had undergone clinically indicated coronary computed tomography angiography (CCTA). The annotated ground truth included the ascending and descending aortas (PAA, DA), superior and inferior vena cavae (SVC, IVC), pulmonary artery (PA), coronary sinus (CS), right ventricular wall (RVW), and left atrial wall (LAW). Figure 3 shows the CT images of dataset_2. Table 2 outlines the description of the datasets. Both datasets contain CT images of CAD and Non-CAD patients.

The study applies the following steps for identifying CAD using CNN architecture from datasets:

Step 1: Preprocess images

The CCTA images are processed to fit the feature extraction phase. All images are converted into 600 × 600 pixels. The image size suits the feature extraction process to generate a reduced set of features without losing any valuable data.

Step 2: Feature extraction

**Figure 4:**
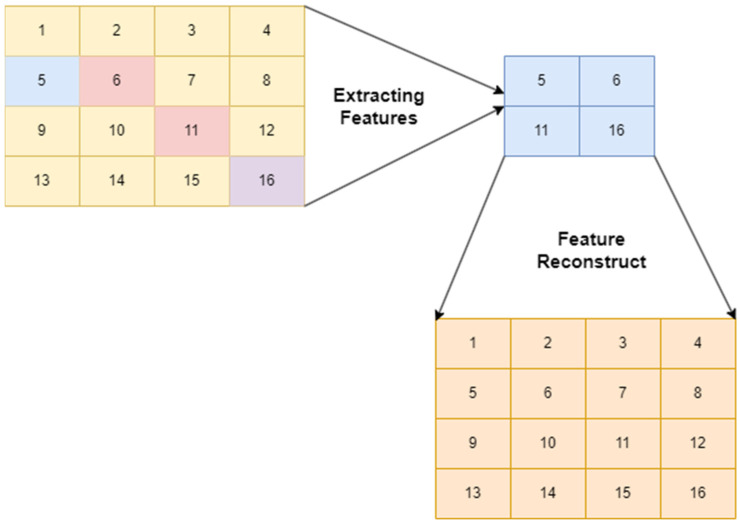
Process of feature extraction.

The proposed study applies an enhanced features from accelerated segment test (FAST) [6] algorithm for extracting features to support the pooling layer of CNN to produce effective feature maps to answer RQ1. To reduce the processing time of the FAST algorithm, researchers employed the enhanced FAST [5]. Figure 4 showcases the feature extracted from a 4 × 4 image into a 2 × 2 image. In addition, it highlights that the actual image can be reconstructed from a 2 × 2 image to a 4 × 4 image.

The extraction process is described as follows:

Let image *I* of *M*_1_ × *M*_2_ pixels be divided into segments *S*_1_ × *S_n_*. The number of segments is *N*_1_ × *N*_2,_ where *N*_1_ = *M*_1_/*S*_1_ and *N*_2_ = *M*_2_/*S_n_*. The segments are represented in Equation (1).

(1) $$I=\left[\begin{array}{ccc} Sd_{1,1} & Sd_{1,2}\ldots & Sd_{1,N_{n}}\\ \vdots & \vdots \; & \vdots \\ Sd_{N_{1},1} & Sd_{N_{1},2\;}\ldots & Sd_{N_{1},N_{n}\;} \end{array}\right]\;$$

where *Sd_x,y_* referred to the image segment in the *x* and *y* direction and is described in Equation (2).

*Sd_x,y_* = *I(i,j)* (2)

where *i* and *j* represent the size of the image segment, *Sdx,y*.

Both Equations (3) and (4) describe the pixel values of image segments.

(3) $$i=\left(y-1\right)M_{2},\left(y-1\right)M_{2}-1,\;\ldots ,\;yM_{2}-1$$

(4) $$j=\left(x-1\right)M_{1},\left(x-1\right)M_{1}-1,\;\ldots ,\;yM_{1}-1$$

The transformation function ensures that the image or segment can be reconstructed to its original form. It supports the proposed method to backtrack the CNN network to fine-tune its performance. The transformation function for each segment is mentioned in Equation (5) as follows:

(5) $$\varphi Md_{x,y}=Z_{S_{1}}Md_{x,y}Z_{M_{2}}^{T}$$

where $\varphi Md_{x,y}$ represents a part of an extracted feature from the image segment, *x* = $1,\ldots \ldots ,N_{1}$, *y* = $1,\ldots \ldots ,N_{n}$ and *T* represents the transform matrix, $Z_{M1}\in Z_{M_{1}}^{O}$, *O* represents the order of the transformation. The segment can be reconstructed as in Equation (6).

(6) $$Sd_{x,y}=Z_{S_{1}}^{T}\varphi Sd_{x,y}Z_{S_{n}}$$

Sequentially, the process must be repeated *N*_1_ × *N_n_* times to extract a set of features from the image. Thus, the transform co-efficient of all image segments can be integrated using Equations (7)–(11).

(7) $$\varphi =\left[\begin{array}{ccc} Z_{S_{1}}Sd_{1,1}Z_{S_{n}}^{T} & \ldots & Z_{S_{1}}Sd_{1,N_{n}}Z_{S_{2}}^{T}\\ \vdots & \ldots & \vdots \\ Z_{S_{1}}Sd_{N_{1},1}Z_{S_{n}}^{T} & \ldots & Z_{S_{1}}Sd_{N_{1},N_{n}}Z_{S_{2}}^{T} \end{array}\right]$$

Equations (8) and (9) denote the features $F_{S_{1}}$ and $F_{S_{n}}$, which represent the features that can be constructed using $Z_{s1}$ & $Z_{sn}$, as follows:

(8) $$F_{S_{1}}=\left[\begin{array}{cccc} Z_{S_{1}} & O & \ldots & O\\ O & Z_{S_{1}} & \ldots & \vdots \\ \ddots & \vdots & \vdots & O\\ O & \ldots & O & Z_{S_{1}} \end{array}\right]\;\mathrm{order}\;\mathrm{of}\;N_{1}$$

(9) $$F_{S_{n}}=\left[\begin{array}{cccc} Z_{S_{n}} & O & \ldots & O\\ O & Z_{S_{n}} & \ldots & \vdots \\ \ddots & \vdots & Z_{S_{n}} & O\\ O & \ldots & O & Z_{S_{n}} \end{array}\right]\;\mathrm{order}\;\mathrm{of}\;N_{n}$$

Equation (10) shows a sample set of features, $\partial_{nm}$.

(10) $$\underset{x\in X}{\sum }\left(F_{S\left(n,x\right)}*F_{S\left(m,x\right)}\right)=\partial_{nm}$$

Equation (11) defines the reconstruction of the image using the extracted features.

(11) $$I=F_{S_{1}}^{T}\;\varphi \;F_{S_{n}}$$

Step 3: Processing features

**Figure 5:**
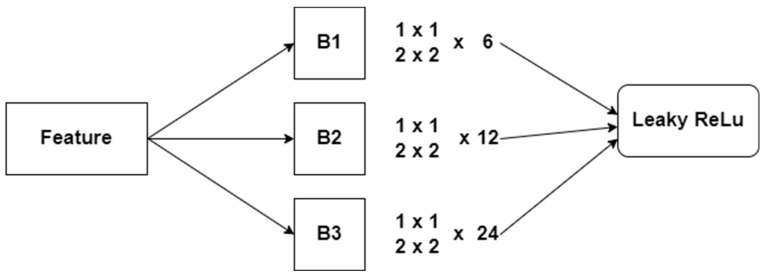
Fine-tuned DenseNet Architecture.

The extracted features $F_{S_{1}}\ldots \ldots \;F_{S_{n}}$ are treated as an input for the proposed CNN. DenseNet ensures the transmission of information between the layers. One of the features of the DenseNet is the direct link between each layer. Thus, a back propagation method can be implemented in DenseNet. The feature extraction process reduces the number of blocks in DenseNet and improves its performance. Therefore, the modified DenseNet contains a smaller number of blocks and parameters. Research studies highlight that the complex network requires a greater number of samples. This study applies DenseNet-161 (K = 48), which includes three block modules. Figure 5 illustrates the proposed DenseNet model. Most CNN models depend on the features to make a decision. Thus, the feature extraction process is crucial in disease detection techniques. The minimal set of features reduces the training time of the CNN model. In addition, the features should support CNN to generate effective results. Researchers applied an edge-detection technique.

Step 3.1: Pooling layer

Two-dimensional filters are used to integrate the features in the area covered by the two-dimensional filter as it slides over each feature map channel. The dimension of the pooling layer output is in Equation (12):

(12) $$(I_{h}-f+1)/l*\left(I_{w}-f+1\right)/s*I_{c}$$

where $I_{h}$—the height of the feature map, $I_{w}$—width of the feature map, $I_{c}$—number of channels in the map, *f*—filter size, *l*—stride length

Step 3.2: Generating output

**Figure 6:**
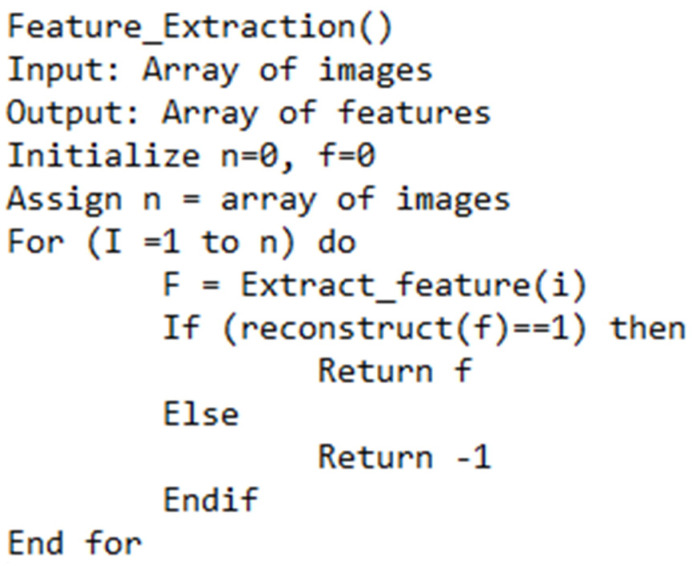
Proposed feature extraction algorithm.

**Figure 7:**
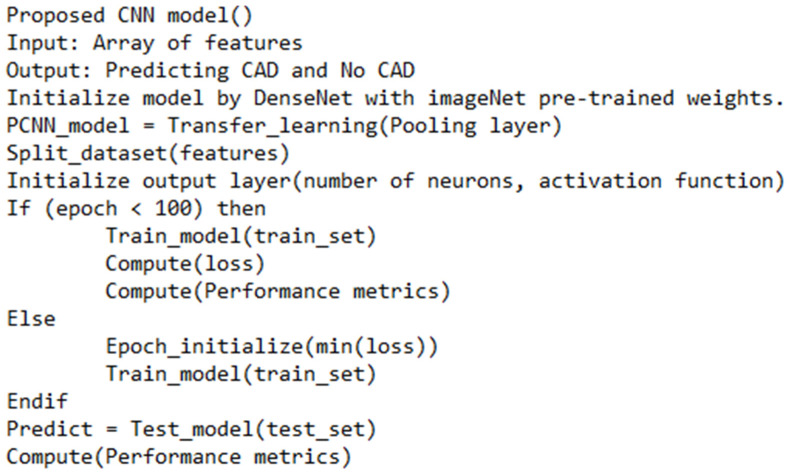
Proposed CNN model.

Transfer learning is adopted to alter the architecture of DenseNet. Leaky ReLu is used as the activation function. The existing CNN includes are employed. GITHUB portal (https://github.com/titu1994/DenseNet accessed on 7 December 2021) is utilized to implement the existing CNN architecture. The studies [10,18,21] are employed to evaluate the performance of the proposed CNN (PCNN) model. In addition, CNN models, including GoogleNet and Inception V3, are used for performance evaluation. The following form of the sigmoid function is applied for implementing the modified DenseNet. Figure 6 represents the proposed feature extraction for pre-processing the CT images and extracting the valuable features. Furthermore, Figure 7 highlights the proposed CNN technique for predicting CAD from the CT images.

The study constructs a feed-forward back propagation network. Thus, Leaky ReLu is employed in the study as an activation function in Equation (13) to produce an outcome.

(13) f(x)=max(0,x)

Leaky ReLu considers negative value as a minimal linear component of X. The definition of Leaky ReLu is defined as:

Def Leaky_function(I)

  If feature(I) < 0:

return 0.01 * f(I)

  Else:

return f(I)

Step 4: Evaluation metrics

The study applies the benchmark evaluation metrics, including accuracy, recall, precision, and F-measure, to provide a solution for RQ2. The metrics are computed as shown in Equations (14)–(18):

True positive (TP_CI_) = predicting a valid positive CAD patient from CT images (CI).

True negative (TN_CI_) = predicting a valid negative CAD patient from CI.

False positive (FP_CI_) = predicting a negative CAD patient as positive from CI.

False negative (FN_CI_) = predicting a positive CAD patient as negative from CI.

(14) $$\mathrm{Recall}=\frac{{\mathrm{TP}}_{\mathrm{CI}}}{{\mathrm{TP}}_{\mathrm{CI}}+{\mathrm{FN}}_{\mathrm{CI}}}$$

(15) $$\mathrm{Precision}=\frac{{\mathrm{TP}}_{\mathrm{CI}}}{{\mathrm{TP}}_{\mathrm{CI}}+{\mathrm{FP}}_{\mathrm{CI}}}$$

(16) $$F-\mathrm{measure}=\frac{2*\mathrm{Recall}*\mathrm{Precision}}{\mathrm{Recall}+\mathrm{Precision}}$$

(17) $$\mathrm{Accuracy}=\frac{{\mathrm{TP}}_{\mathrm{CI}}+{\mathrm{TN}}_{\mathrm{CI}}}{{\mathrm{TP}}_{\mathrm{CI}}+{\mathrm{TN}}_{\mathrm{CI}}+{\mathrm{FP}}_{\mathrm{CI}}+{\mathrm{FN}}_{\mathrm{CI}}}$$

(18) $$\mathrm{Specificity}=\frac{{\mathrm{TN}}_{\mathrm{CI}}}{{\mathrm{TN}}_{\mathrm{CI}}+{\mathrm{FP}}_{\mathrm{CI}}}$$

In addition, Matthews correlation coefficient (MCC) (Equation (19)) and Cohen’s Kappa (K) (Equation (20)) are employed to ensure the performance of the proposed method.

(19) $$\mathrm{MCC}=\frac{\left({\mathrm{TP}}_{\mathrm{CI}}{*\mathrm{TN}}_{\mathrm{CI}}\right)-\left({\mathrm{FP}}_{\mathrm{CI}}{*\mathrm{FN}}_{\mathrm{CI}}\right)}{\sqrt{\;\left({\mathrm{TP}}_{\mathrm{CI}}+{\mathrm{FP}}_{\mathrm{CI}}\right)*\left({\mathrm{TP}}_{\mathrm{CI}}+{\mathrm{FN}}_{\mathrm{CI}}\right)*\left({\mathrm{TN}}_{\mathrm{CI}}+{\mathrm{FP}}_{\mathrm{CI}}\right)*\left({\mathrm{TN}}_{\mathrm{CI}}+{\mathrm{FN}}_{\mathrm{CI}}\right)}}$$

The minimum MCC is −1, which indicates a wrong prediction, whereas the maximum MCC is +1, which denotes a perfect prediction.

(20) $$K=\frac{2*\left(\left({\mathrm{TP}}_{\mathrm{CI}}{*\mathrm{TN}}_{\mathrm{CI}}\right)-\left({\mathrm{FP}}_{\mathrm{CI}}{*\mathrm{FN}}_{\mathrm{CI}}\right)\right)}{\left({\mathrm{TP}}_{\mathrm{CI}}+{\mathrm{FP}}_{\mathrm{CI}}\right)*\left({\mathrm{FP}}_{\mathrm{CI}}+{\mathrm{TN}}_{\mathrm{CI}}\right)*\left({\mathrm{TP}}_{\mathrm{CI}}+{\mathrm{FN}}_{\mathrm{CI}}\right)*\left({\mathrm{FN}}_{\mathrm{CI}}+{\mathrm{TN}}_{\mathrm{CI}}\right)}$$

MCC and K are class symmetric, reflecting the ML technologies’ classification accuracy. Finally, CNN technique computational complexity is presented to find the time and space complexities.

In order to ensure the predictive uncertainty of the proposed CNN (PCNN), the researchers applied standard deviation (SD) and entropy (E). The mathematical expression of the confidence interval (CI) is defined in Equation (21).

(21) $$\mathrm{CI}=a\pm z\frac{\sigma }{\sqrt{N}}$$

where *a* represents the mean of the predictive distribution of an image $a^{\left(i\right)}$, *N* is the total number of predictions, and *z* is the critical value of the distribution. The researchers computed CI at 95% confidence. Thus, the value of *Z* is 1.96.

Finally, the researchers followed *E* of the prediction to evaluate the uncertainty of the proposed model. It is calculated over the mean predictive distribution. The mathematical expression of *E* is defined in Equation (22).

(22) $$E(P\left(y^{*}|a^{*}\right)=-\sum_{i=1}^{C}P\left(y^{*}|a^{*}\right)\log(P\left(y^{*}|a^{*}\right)$$

## 4. Experiment and Results

The PCNN is implemented in Python with Windows 10 Professional platform. The existing algorithms are developed using the GITHUB portal. Both datasets are divided into training and testing sets. Accordingly, the CNN architectures are trained with a relevant training set of dataset_1 and dataset_2.

To evaluate the performance of PCNN, the dataset is utilized using 5-fold cross-validation. Statistical tests, including SD, CI using binary class classification, and E are applied accordingly on the dataset_1 and dataset_2. Table 3 presents the implementation of PCNN during the cross-validation using daaset_1. It highlights that PCNN achieves more than 98% accuracy, precision, recall, F-measure, and specificity, respectively. Likewise, Table 4 denotes the cross-validation outcome for dataset_2.

### 4.1. Uncertainty Estimation

In this study, the researchers apply Monte Carlo dropout (MC dropout) to compute the model uncertainty. The dropout value ensures that the predictive distribution is not diverse, and CI is insignificant. The researchers experimentally found that the MC dropout value of 0.379 is optimal for this model. The predictive distribution is obtained by evaluating PCNN 200 times for each image. Furthermore, model uncertainty is computed using CI, SD, and E.

Table 5 and Table 6 highlight the model uncertainty for dataset_1 and dataset_2, respectively. The proposed model achieved a low entropy and SD for both datasets. It can be observed in Table 5 and Table 6 that the average CI of [98.55–98.61] and [98.45–98.51] for dataset_1 and dataset_2 indicate the proposed model has high confidence and minimum variance in its outcome.

**Figure 8:**
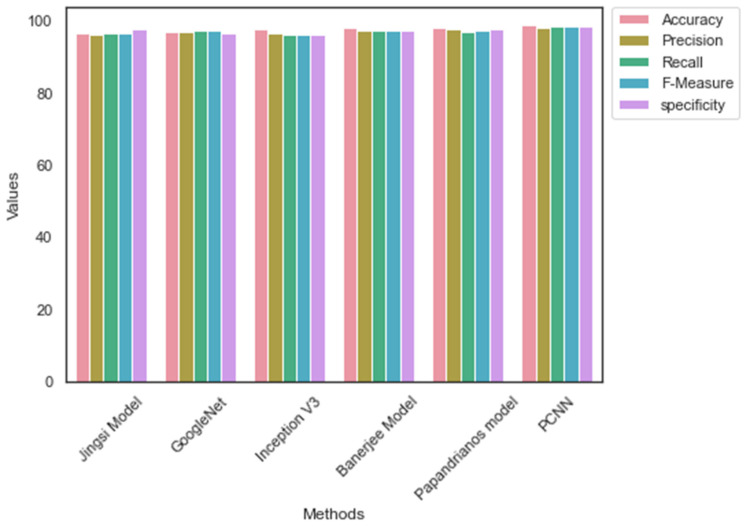
Comparative analysis outcome: Dataset_1.

Table 7 highlights the performance measures of dataset_1. Among the CNN architectures, PCNN scored a superior accuracy, precision, recall, and specificity of 98.96, 98.2, 98.52, 98.36, and 98.7, respectively. The performance of the Banerjee model [18] is lower than the other CNN architectures. PCNN performs better than the existing CNN models for CAD prediction. Dataset_1 contains a greater number of images. The mapping of features made the CNN architectures generate more features. However, the feature extraction process of the proposed method enabled PCNN to produce a smaller number of features and maintain a better performance than the existing architectures. Figure 8 represents the comparative analysis outcome of CNN. It is evident from Figure 8 that the performance of PCNN is higher than the current architectures.

**Figure 9:**
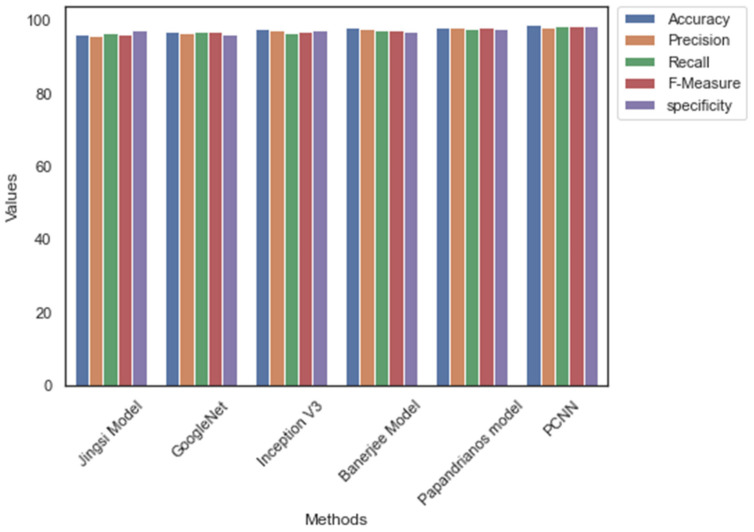
Comparative analysis outcome: Dataset_1.

Likewise, Table 8 outlines the performance of CNN architectures with Dataset_2. The value of accuracy, precision, recall, F-measure, and specificity is 98.96, 98.2, 98.52, 98.36, and 98.7, accordingly. However, GoogleNet has scored low accuracy, precision, recall, F-measure, and specificity of 97.1, 96.7, 97.1, 96.9, and 96.4, respectively. The absence of temporary memory is one of the limitations of the Banerjee model that reduces its predicting performance. In addition, the outcome of Table 5 and Table 6 suggest that the performance of PCNN is higher than the existing CNN architectures. Figure 9 shows the relevant graph of Table 6.

**Figure 10:**
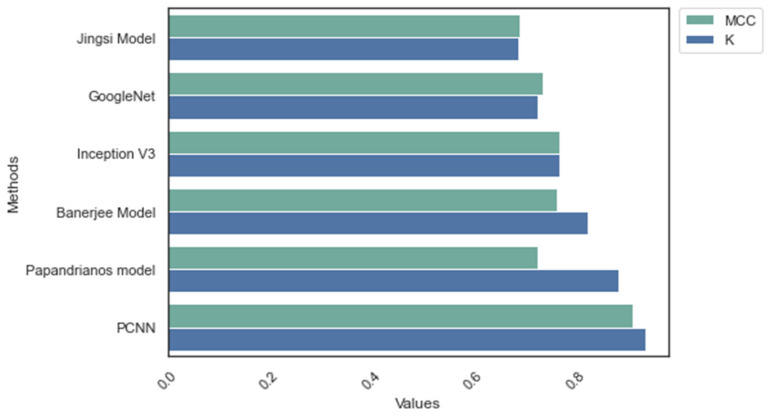
MCC and Kappa: Dataset_1.

**Figure 11:**
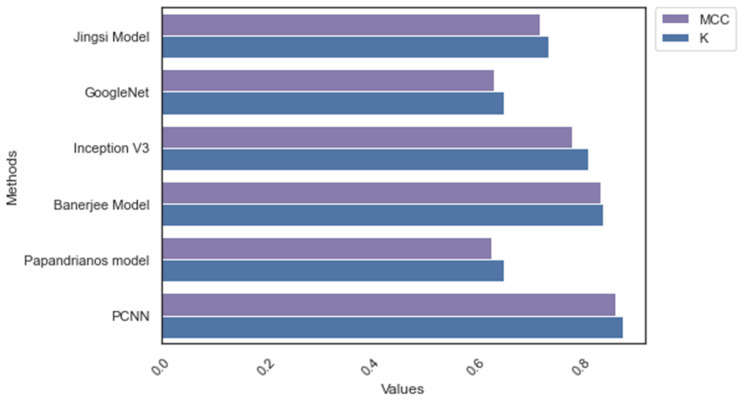
MCC and Kappa: Dataset_2.

In addition to the initial comparative analysis, the researcher applied MCC and Kappa to evaluate the performance of PCNN. Figure 10 and Figure 11 reveal that PCNN achieved a superior MCC and K score compared to the existing models.

**Figure 12:**
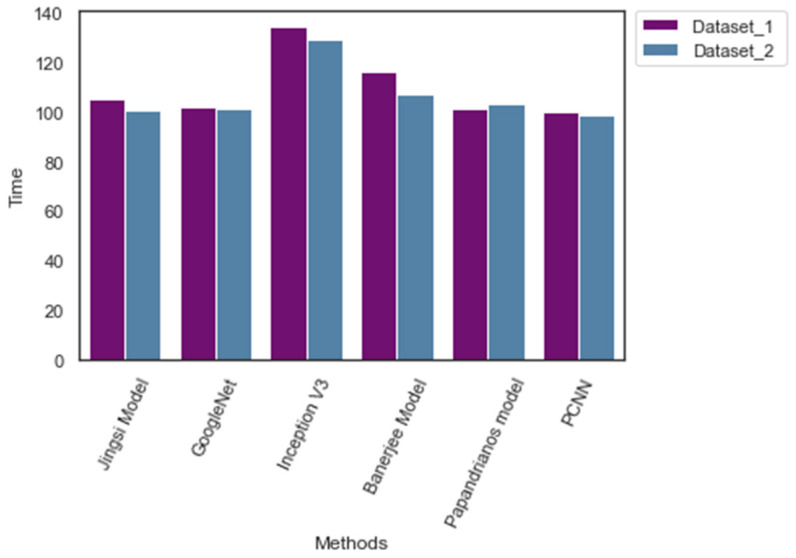
Computation time of CNN models.

Table 9 outlines the memory size and computing time during the training phase. PCNN consumes 121.45 MB and 118.45 MB for Dataset_1 and Dataset_2, accordingly. The computing time of PCNN is 99.32 min and 99.21 min, respectively. The computing time of PCNN is superior to the existing CNN with less memory. Figure 12 highlights CNN’s space and computation time for both Dataset_1 and Dataset_2.

**Figure 13:**
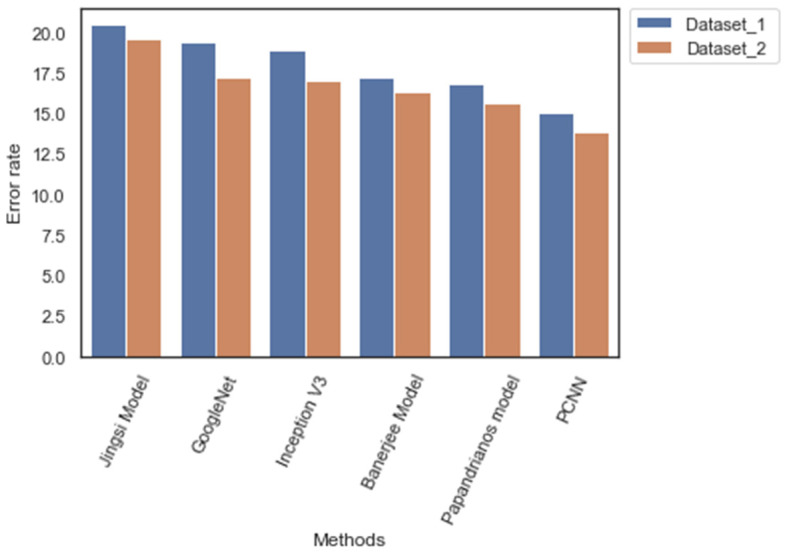
Error rates of CNN models.

Table 10 outlines the error rate of the CNN architectures during the testing phase. The error rate of PCNN is 15.1 and 13.9 for Dataset_1 and Dataset_2, respectively. Nevertheless, Jingsi model scores 20.5 and 19.6, which is higher than other CNN models. The outcome emphasizes the efficiency of the feature extraction process of PCNN. Figure 13 illustrates the error rate of CNN models.

**Figure 14:**
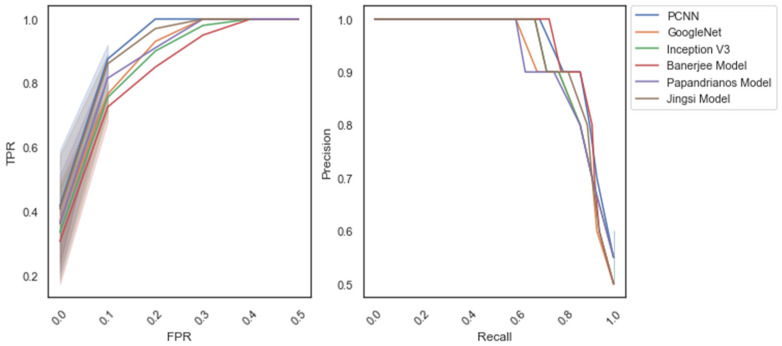
Receiver operating characteristic (ROC) and precision–recall curve: dataset_1.

Figure 14 represents the receiver operating characteristic (ROC) and precision–recall (PR) curve for dataset_1 during the testing phase. It shows that PCNN achieves a better Area under the ROC curve (AUC) for CAD and No CAD classification, respectively.

**Figure 15:**
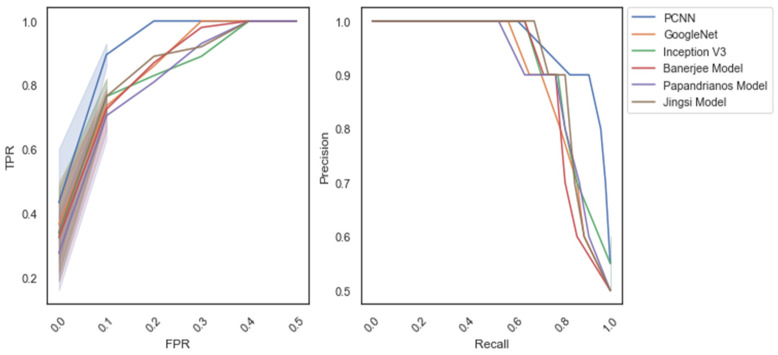
Receiver operating characteristic (ROC) and precision–recall curve: dataset_2.

Similarly, Figure 15 reflects the ROC and PR curve for dataset_2. It outlines that PCNN achieves a better ROC AUC score of 0.93. Furthermore, the AUC score of the PR curve (0.91) indicates that PCNN predicts CAD better than the existing models.

Table 11 highlights the computational complexities of CNN models for Dataset_1. It is evident from the outcome that PCNN requires a smaller number of parameters (4.3 M), learning rate (1 × 10^−4^), number of flops (563 M), and computation time (1.92 s).

Likewise, Table 12 reflects the outcome for Dataset_2. It shows that PCNN generates an output with fewer parameters, flops, and learning rates than the existing CNN models.

### 4.2. Clinical Insights and Limitations

PCNN generates outcomes that are superior to the existing CNN models. It can be employed in real-time applications to support physicians in diagnosing CAD. In addition, it can be integrated with Internet of Things devices to support healthcare centers in identifying CAD at an earlier stage. The feature extraction and the pooling layer of PCNN can detect CAD from complex CT images. The dropout layer reduces the neurons to avoid limitations such as overfitting and underfitting. PCNN applies loss function to compute the kernels and weights of the model. It optimizes the model’s performance and generates a meaningful outcome.

PCNN produces an effective result and supports CAD diagnosing process. However, a few limitations need to be addressed in future studies. The multiple layers of CNN increase the training time and require a better graphical processing unit. The imbalanced dataset may reduce the performance of the proposed method. The researcher introduced the concept of temporary storage to hold the intermediate results.

Nonetheless, there is a possibility of losing information due to multiple features. The lack of co-ordinate frames may lead to the adversarial visualization of images. The feature selection process can improve the images’ internal representation. Finally, the structure of PCNN requires a considerable amount of data to produce an exciting result. To maintain the better performance, data pre-processing is necessary to handle image rotation and scaling tasks.

## 5. Conclusions

This study developed a CNN model for predicting CAD from CT images. The existing CNN architectures require a high-end hardware configuration for processing complex images. A feature extraction technique is employed to support the proposed CNN model. The proposed method modifies the existing DenseNet architecture in order to implement a feed-forward back-propagation network. Two benchmark datasets are used for the performance evaluation. The experiment analysis’s outcome highlights the superior performance of the proposed CNN model in terms of accuracy, precision, recall, F-measure, and specificity. Moreover, the proposed CNN’s memory consumption and computation time during the training phase are lower than the existing CNNs. In addition, ROC and PR curve analysis suggest that the proposed method can predict CAD with a lower false positive rate with higher prediction accuracy. Thus, the proposed method can support the physician in detecting and preventing CAD patients. In the future, the proposed model can be implemented to predict CAD from electronic health records.

## Figures and Tables

**Table 1:** Features of the existing literature.

Authors	Methodology	Features	Limitations
Lin. S et al. [1]	Conducted a cross-sectional study of CAD patients for validating CNN-based CAD.	The findings showed that the deep learning algorithm could support physicians in detecting cardiovascular diseases.	The findings are based on the specific location and lack of a benchmark dataset for evaluating the CNN model.
Jingsi Z et al. [10]	Proposed a low-light image enhancement method.	The DenseNet framework has reduced the noise in the images.	Lack of discussion of the application of bright images.
Abdar M et al. [13]	Integrated genetic algorithm and support vector machine for feature extraction.	The outcome showed that N2Genetic-nuSVM showed a better accuracy.	Lack of comparison with the recent techniques.
Wolterink J.M. et al. [20]	A 3D-dilated CNN is developed to predict the radius of an artery from CCTA images.	Results show that the method extracted 92% of clinically relevant coronary artery segments.	Trained with a small dataset. The outcome may be with the size of the dataset.
Papandrianos N. and Papageorgiou E. [21]	Applied CNN model for CAD detection from images.	The method can differentiate the infarction from healthy patients.	The classification accuracy is better. However, there is a lack of benchmark evaluation techniques.
Nishi et al. [27]	Developed an image segmentation technique for predicting CAD.	The outcome highlighted that the method could produce effective results.	The performance is based on a single dataset.
Cho et al. [30]	Proposed an intravascular ultrasound-based algorithm for classifying attenuation and calcified plaques.	The results outlined that the model achieved 98% accuracy.	The model performance is based on the dataset of 598 patients.
Morris S.A. and Lopez K.N. [31]	Developed a detection model for congenital heart disease in the fetus.	The outcome showed that the model’s performance is better than the recent models.	The authors evaluated the model using 1326 fetal echocardiograms.
Cheung et al. [36]	Proposed an image segmentation approach using Unet model.	The model achieved 91,320% of dice similarity coefficient.	The lack of discussion of the image quality used in the study.
Bhanu Prakash Doppala et al. [37]	Developed an ensemble model for cardiovascular disease detection.	The model achieves an accuracy of 96.75%.	The model is based on the voting mechanisms, which may lead to a larger computation time.
Ali Md Mamun et al. [38]	Proposed an ML algorithm for heart disease detection.	The outcome shows that the model has achieved a 100% of accuracy with the Kaggle dataset.	There is a lack of experimentation with the model with different datasets.
Khanna, Ashish et al. [39]	Developed an ML technique for heart disease detection from ECG.	Employed regression model to predict heart disease from ECG.	Limited discussion on the model uncertainty.
Yan, Jielu et al. [40]	Proposed an ML technique for predicting ion channel peptides.	The outcome shows that the model achieves high accurate results.	The dataset is relatively small.

**Table 2:** Description of datasets.

Dataset	Number of Patients	Number of Images	Classification
1	500	2637	2
2	200	716	2

**Table 3:** Performance analysis of PCNN model for dataset_1.

Fold(s)	Accuracy	Precision	Recall	F-Measure	Specificity
1	98.6	97.4	98.4	97.9	98.5
2	98.2	98.2	97.9	98.05	97.8
3	99.1	97.7	98.3	98	98.8
4	99.3	98.6	98.7	98.65	98.8
5	99.6	99.1	99.3	99.2	99.6
Average	98.96	98.2	98.52	98.36	98.7

**Table 4:** Performance analysis of PCNN model for dataset_2.

Fold(s)	Accuracy	Precision	Recall	F-Measure	Specificity
1	98.4	97.8	98.2	98	98.1
2	97.8	99.3	99.1	99.2	99.3
3	99.1	98.7	98.7	98.7	98.6
4	98.9	98.2	98.6	98.4	98.2
5	99.1	99.3	98.7	99	98.9
Average	98.66	98.66	98.66	98.66	98.62

**Table 5:** Model uncertainty analysis outcome for dataset_1.

Fold(s)	CI (%) @95%	SD	Entropy
1	[97.92–97.99]	0.0012	0.0049
2	[98.12–98.19]	0.0019	0.0329
3	[98.79–98.87]	0.0021	0.0319
4	[98.84–98.91]	0.0020	0.0281
5	[99.08–99.11]	0.0017	0.0091
Average	[98.55–98.61]	0.0017	0.0213

**Table 6:** Model uncertainty analysis outcome for dataset_1.

Fold(s)	CI (%) @95%	SD	Entropy
1	[98.11–98.18]	0.0021	0.0041
2	[97.41–97.49]	0.0018	0.0312
3	[98.42–98.46]	0.0014	0.0187
4	[99.12–99.17]	0.0011	0.0093
5	[99.21–99.26]	0.0009	0.0089
Average	[98.45–98.51]	0.0014	0.0144

**Table 7:** Comparative analysis outcome of CNN model for dataset_1.

Methods/ Measures	Accuracy	Precision	Recall	F-Measure	Specificity
Jingsi model [10]	96.7	96.2	96.7	96.45	97.65
GoogleNet	96.9	97.1	97.4	97.25	96.5
Inception V3	97.8	96.7	96.1	96.4	96.2
Banerjee model [18]	98.1	97.3	97.5	97.4	97.57
Papandrianos model [21]	98.3	97.6	97.1	97.35	97.69
PCNN	98.96	98.2	98.52	98.36	98.7

**Table 8:** Comparative analysis outcome of CNN model for dataset_2.

Methods/ Measures	Accuracy	Precision	Recall	F-Measure	Specificity
Jingsi model	96.3	95.8	96.7	96.25	97.2
GoogleNet	97.1	96.7	97.1	96.9	96.4
Inception V3	97.6	97.2	96.8	97	97.3
Banerjee model	98.1	97.6	97.5	97.55	97.1
Papandrianos model	98.3	98.2	97.9	98.05	97.8
PCNN	98.96	98.2	98.52	98.36	98.7

**Table 9:** Memory sizes of CNN for Dataset_1 and Dataset_2.

Methods/Datasets	Dataset_1 (MB)	Dataset_2 (MB)	Dataset_1 Time (Minutes)	Dataset_2 Time (Minutes)
Jingsi model	279.21	189.32	105.26	101.25
GoogleNet	175.69	159.27	102.26	101.36
Inception V3	138.14	142.58	134.56	129.71
Banerjee model	128.54	143.96	116.32	107.25
Papandrianos model	129.65	137.89	101.45	103.59
PCNN	119.25	124.26	100.56	98.89

**Table 10:** Error rates of CNN for Dataset_1 and Dataset_2.

Methods/Measures	Dataset_1 (%)	Dataset_2 (%)
Jingsi model	20.5	19.6
GoogleNet	19.4	17.3
Inception V3	18.94	17.1
Banerjee model	17.3	16.4
Papandrianos model	16.9	15.7
PCNN	15.1	13.9

**Table 11:** Computational complexities of CNN for Dataset_1.

Methods/Measures	Number of Parameters	Learning Rate	Number of Flops	Testing Time (s)
Jingsi model	5.1 M	1 × 10^−3^	565 M	2.5
GoogleNet	6.7 M	1 × 10^−3^	624 M	2.36
Inception V3	7.4 M	1 × 10^−4^	594 M	2.7
Banerjee model	14.6 M	1 × 10^−3^	1421 M	2.3
Papandrianos model	11.2 M	1 × 10^−2^	1530 M	2.1
PCNN	4.3 M	1 × 10^−4^	563 M	1.92

**Table 12:** Computational complexities of CNN for Dataset_2.

Methods/Measures	Number of Parameters	Learning Rate	Number of Flops	Computation Time (s)
Jingsi model	4.3 M	1 × 10^−3^	436 M	1.91
GoogleNet	5.6 M	1 × 10^−3^	512 M	1.72
Inception V3	6.3 M	1 × 10^−5^	402 M	1.86
Banerjee model	9.4 M	1 × 10^−4^	921 M	1.98
Papandrianos model	10.3 M	1 × 10^−3^	430 M	1.36
PCNN	3.7 M	1 × 10^−5^	403 M	1.15

## Data Availability

The data presented in this study are available on request from the corresponding author.
